# Prognostic biomarker *SGSM1* and its correlation with immune infiltration in gliomas

**DOI:** 10.1186/s12885-022-09548-7

**Published:** 2022-04-28

**Authors:** Junsheng Li, Jia Wang, Yaowei Ding, Jizong Zhao, Wen Wang

**Affiliations:** 1 Department of Neurosurgery, Beijing Tiantan Hospital, Capital Medical University, Nan Si Huan Xi Road 119, Fengtai District, Beijing, 100070 China; 2 China National Clinical Research Center for Neurological Diseases, Beijing, China; 3 Center of Stroke, Beijing Institute for Brain Disorders, Beijing, China; 4 Beijing Key Laboratory of Translational Medicine for Cerebrovascular Disease, Beijing, China; 5 Beijing Translational Engineering Center for 3D Printer in Clinical Neuroscience, Beijing, China; 6 Department of Clinical Diagnosis, Laboratory of Beijing Tiantan Hospital, Capital Medical University, Beijing, China; 7 Savaid Medical School, University of the Chinese Academy of Sciences, Beijing, China

**Keywords:** *SGSM1*, Lower-grade glioma, Immune infiltration, Prognosis, Biomarker

## Abstract

**Objective:**

Glioma was the most common type of intracranial malignant tumor. Even after standard treatment, the recurrence and malignant progression of lower-grade gliomas (LGGs) were almost inevitable. The overall survival (OS) of patients with LGG varied widely, making it critical for prognostic prediction. Small G Protein Signaling Modulator 1 (*SGSM1*) has hardly been studied in gliomas. Therefore, we aimed to investigate the prognostic role of *SGSM1* and its relationship with immune infiltration in LGGs.

**Methods:**

We obtained RNA sequencing data from The Cancer Genome Atlas (TCGA) to analyze *SGSM1* expression. Functional enrichment analyses, immune infiltration analyses, immune checkpoint analyses, and clinicopathology analyses were performed. Univariate and multivariate Cox regression analyses were used to identify independent prognostic factors. And nomogram model has been developed. Kaplan–Meier survival analysis and log-rank test were used to estimate the relationship between OS and *SGSM1* expression. The survival analyses and Cox regression were validated in datasets from the Chinese Glioma Genome Atlas (CGGA).

**Results:**

*SGSM1* was significantly down-regulated in LGGs. Functional enrichment analyses revealed *SGSM1* was correlated with immune response. Most immune cells and immune checkpoints were negatively correlated with *SGSM1* expression. The Kaplan–Meier analyses showed that low *SGSM1* expression was associated with a poor outcome in LGG and its subtypes. The Cox regression showed *SGSM1* was an independent prognostic factor in patients with LGG (HR = 0.494, 95%CI = 0.311–0.784, *P* = 0.003).

**Conclusion:**

*SGSM1* was considered to be a new prognostic biomarker for patients with LGG. And our study provided a potential therapeutic target for LGG treatment.

## Introduction

Gliomas were the most common primary intracranial malignant tumors which originated from glial cells [1–3]. According to the World Health Organization (WHO) grading system, grade II and III gliomas were classified as lower-grade gliomas (LGGs) [4–6]. The median overall survival (OS) of grade II and III glioma patients were 78.1 months and 37.6 months, respectively [7]. Although LGG was a more indolent precursor to glioblastoma (GBM) and less invasive, it caused considerable morbidity and raised a difficult challenge for therapy due to the heterogeneity of clinical behavior [8, 9]. The complete resection of LGG was considered to be still impossible due to the invasive nature. Despite the use of radiotherapy and chemotherapy, local recurrence and progress into GBM were almost inevitable, which led to the decrease in therapeutic effect and a poor prognosis [10–12]. Therefore, prognostic biomarkers were explored to provide a prediction on patients’ survival and response to individualized therapy.

Small G Protein Signaling Modulator 1 (*SGSM1*), located on chromosome 22q11.2, was found to mainly express in brain tissue [13]. Previous research showed the strong association of *SGSM1* with neuronal function. *SGSM1* protein was localized in the trans-Golgi network. Furthermore, *SGSM1* protein possessed RUN domain and TBC domain which was associated with RAP and RAB-mediated cellular signaling. *SGSM1* mediated the interaction between intracellular signaling pathways and vesicle transportation. A recent study has found that *SGSM1* degradation led to the invasion and metastasis of nasopharyngeal carcinoma [14]. Another parallel sequencing research has shown that *SGSM1* was a potential candidate gene for schwannomatosis [15]. However, the role of *SGSM1* has hardly been studied and its prognostic value in LGGs remained unclear.

The data was obtained from TCGA. We investigated the expression patterns of *SGSM1* in LGGs and evaluated its prognostic value. *SGSM1* was down-regulated with the increase of glioma grades, and its low expression indicated a poor prognosis in LGG patients. Moreover, *SGSM1* was associated with immune responses which provided a new sight for personalized treatment. Therefore, *SGSM1* could be a prognostic indicator and a potential therapeutic target for LGGs.

## Method

### RNA-sequencing data acquisition

We downloaded the pan-cancer RNA-seq data of TCGA and GTEx conducted by Toil process uniformly from UCSC XENA (https://xenabrowser.net/datapages/) [16, 17]. For further analyses, we obtained level 3 HTSeq-FPKM and HTSeq-Count data of 529 LGG samples from the TCGA database (https://portal.gdc.cancer.gov/). This study was entirely following the publication guidelines provided by TCGA and GTEx.

### Differential expression gene (DEG) analysis

The median *SGSM1* expression was regarded as the cut-off value to identify DEGs between the two groups (low- and high-expression) of *SGSM1* in LGG samples (HTseq-Count), and we used the DESeq2 R package (1.26.0) for analysis [18].

### Functional enrichment analysis

The threshold of DEGs performed for functional enrichment analysis was defined for |logFC| over 2 and adjusted P-value less than 0.05. Gene Ontology (GO) comprising of biological process (BP), cellular component (CC), and molecular function (MF), as well as Kyoto Encyclopedia of Genes and Genomes (KEGG) analyses were implemented with ClusteProfiler R package (3.14.3) [19, 20].

### Gene set enrichment analysis (GSEA)

We used ClusteProfiler R package (3.14.3) to explore the functional and pathway differences between the two groups of different *SGSM1* expression [21]. For each analysis, the permutation number was set to 1000 times. Enrichment results met the conditions of p.adj < 0.05 and FDR q-value < 0.25 were defined to be statistically significant.

### Immune infiltration and immune checkpoint analyses

We conducted the immune infiltration analysis of *SGSM1* by single-sample Gene Set Enrichment Analysis (ssGSEA) with the GSVA R package (1.34.0) [22]. As mentioned previously, 24 types of infiltrating immune cells were included for analyses [23]. Then we further analyzed the correlation between *SGSM1* and immune checkpoints, including PD1, PD-L1, CTLA4, LAG3, TIM3, TIGIT, and CD48 [24].

### Prognostic model development

We performed univariate and multivariate Cox regression analyses to evaluate whether *SGSM1* could be used as an independent prognostic factor. We have involved clinical parameters, including age, gender, WHO grade, IDH status, and 1p/19q codeletion. Furthermore, nomogram and calibration plot were generated by the RMS package (version 6.2–0) and survival package (version 3.2–10) for predicting 1-year, 3-year, and 5-year OS [2, 25]. We have included the same variables as the Cox regression analyses. The calibration plot has been graphically evaluated by mapping the probabilities predicted by nomogram to observed rates. The diagonal was used as the best predictive value. Concordance index (C-index) was used to determine the discrimination. And the bootstrap method was used to calculate 1000 resamples [26]. In addition, receiver operating characteristic (ROC) curve was used to evaluate the predictive accuracy of the nomogram.

### Validation for survival analyses

Gene expression data and clinicopathological information of 625 LGG samples were retrieved from two RNA-sequencing datasets of CGGA database (http://www.cgga.org.cn/) [27]. It was selected as the validation set to verify the survival analyses and prognostic role of *SGSM1*.

### Statistical analyses

All the statistical analyses and graphs were conducted by the R programming language (version 3.6.3). The expression of *SGSM1* was analyzed by Wilcoxon rank-sum test in unpaired samples. Cox regression analyses assessed the hazard ratios (HRs) and 95% confidence intervals (CIs) of different clinical characteristics, and identified independent prognostic factors. Kaplan–Meier survival analyses and log-rank tests were used to estimate the survival distributions. A two-sided P value less than 0.05 was set to be statistically significant.

## Result

### The expression of *SGSM1* in pan-cancers and LGG

**Fig. 1:**
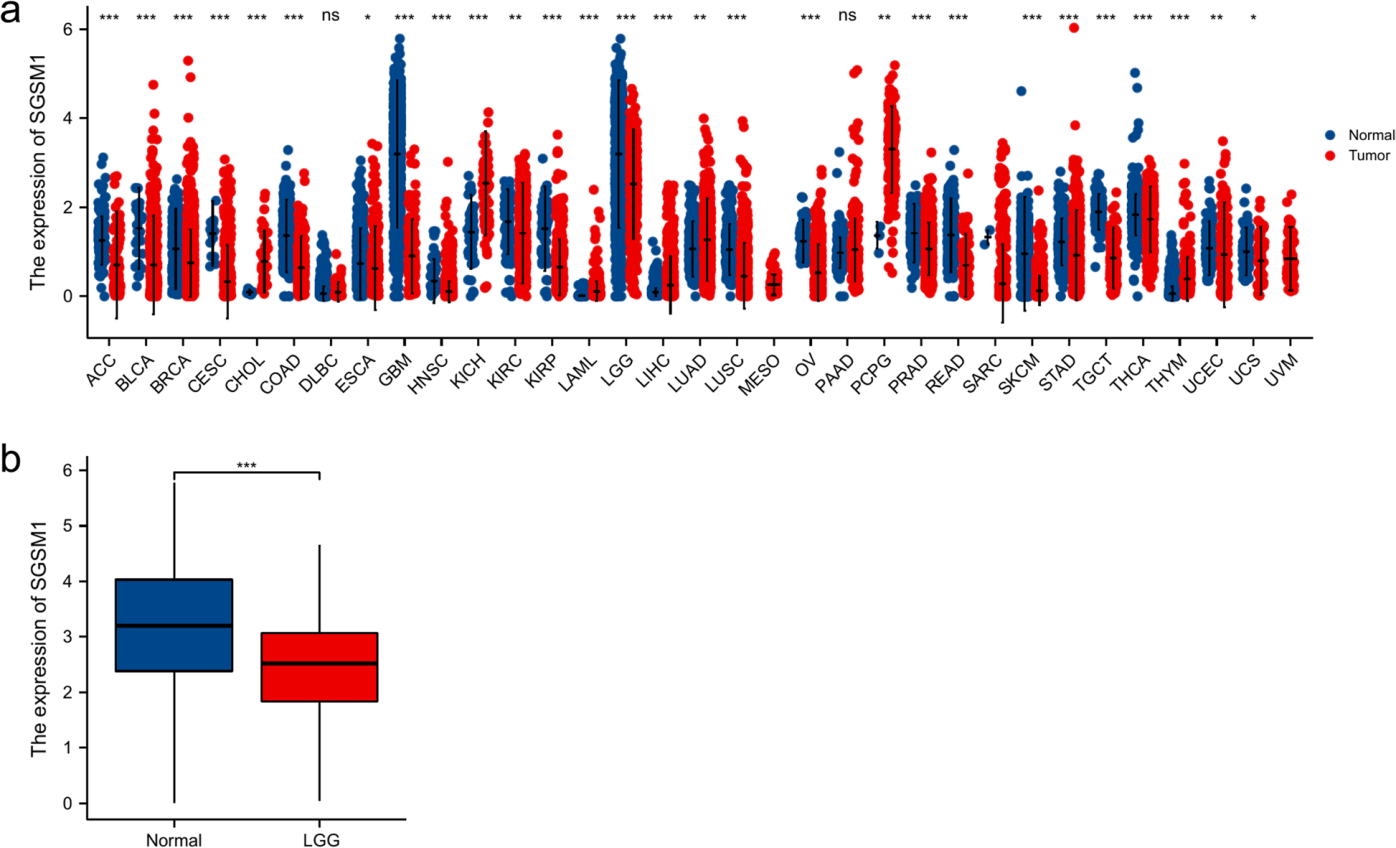
The expression pattern of *SGSM1* in different samples. *P < 0.05; **P < 0.01; ***P < 0.001. **a** *SGSM1* expression between normal tissues and pan-cancer samples; (**b**) *SGSM1* expression between normal tissues and LGGs

Comparing *SGSM1* expression between normal tissues and tumor samples from TCGA and GTEx databases, we found that *SGSM1* was significantly down-regulated in most types of cancer (Fig. 1a), including LGG (P < 0.001, Fig. 1b).

### Identification of DEGs with *SGSM1* and functional enrichment analyses

**Fig. 2:**
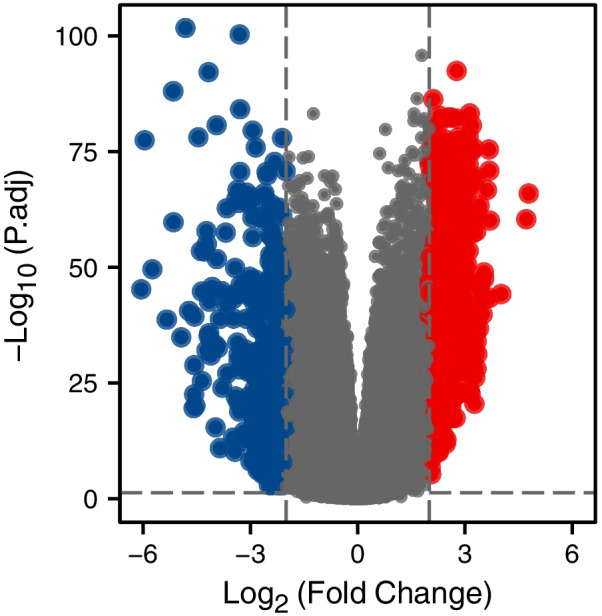
A total of 454 up-regulated and 382 down-regulated genes were identified as being statistically significant between *SGSM1* high expression and low expression groups

A total of 836 DEGs were identified between two groups (low- and high-expression) of *SGSM1* with the criterion of |logFC|> 2 and Padj < 0.05, including 454 up-regulated and 382 down-regulated genes (Fig. 2).

**Fig. 3:**
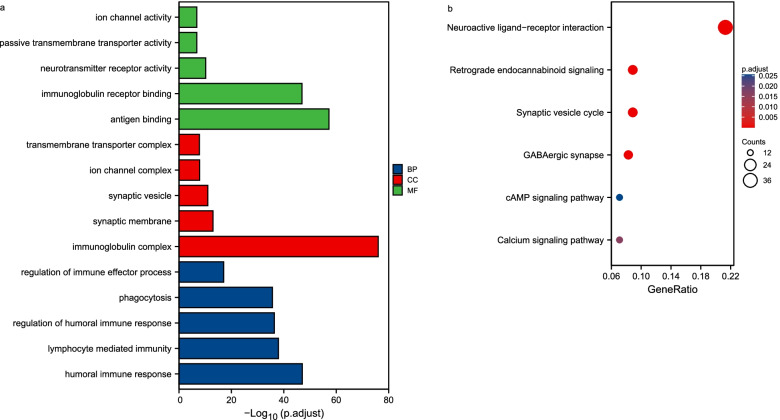
Functional enrichment analyses. **a** GO enrichment analysis; BP biological process, CC cellular component, MF molecular function. **b** KEGG pathway annotation [20]

The results of GO functional analysis and KEGG enrichment analysis have been shown below. BP included humoral immune response, lymphocyte mediated immunity, regulation of humoral immune response, phagocytosis, and regulation of immune effector process. CC included immunoglobulin complex, synaptic membrane, synaptic vesicle, ion channel complex, and transmembrane transporter complex. MF included antigen binding, immunoglobulin receptor binding, neurotransmitter receptor activity, passive transmembrane transporter activity, and ion channel activity (Fig. 3a). KEGG included neuroactive ligand-receptor interaction, retrograde endocannabinoid signaling, synaptic vesicle cycle, GABAergic synapse, cAMP signaling pathway, and calcium signaling pathway (Fig. 3b).

**Fig. 4:**
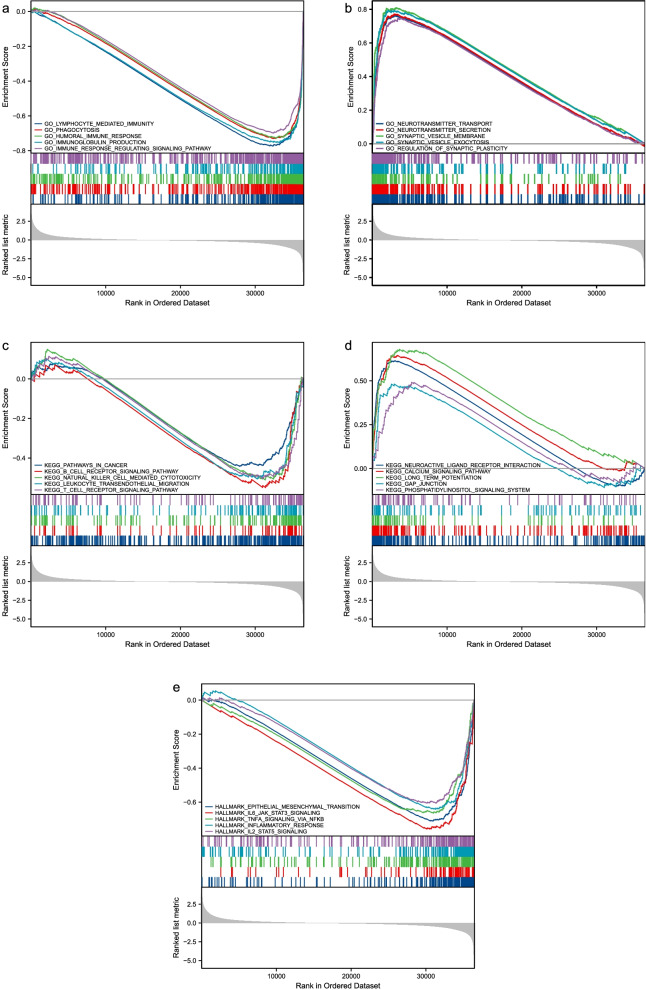
Enrichment analyses from GSEA (**A**-**E**)

We performed GSEA analysis for further identification in biological functions involved in LGGs with different *SGSM1* expression level using the MSigDB collection. Among the significantly enriched gene sets, five GO categories, including lymphocyte mediated immunity, phagocytosis, humoral immune response, immunoglobulin production, and immune response regulating signaling pathway, showed significantly differential enrichment in *SGSM1* low expression phenotype (Fig. 4a); five GO categories, including neurotransmitter transport, neurotransmitter secretion, synaptic vesicle membrane, synaptic vesicle exocytosis, and regulation of synaptic plasticity, showed significantly differential enrichment in *SGSM1* high expression phenotype (Fig. 4b). Five KEGG categories, including pathways in cancer, B cell receptor signaling pathway, natural killer cell mediated cytotoxicity, leukocyte transendothelial migration, and T cell receptor signaling pathway, showed significantly differential enrichment in *SGSM1* low expression phenotype (Fig. 4c); five KEGG categories, including neuroactive ligand receptor interaction, long term potentiation, calcium signaling pathway, gap junction, and phosphatidylinositol signaling system, showed significantly differential enrichment in *SGSM1* high expression phenotype (Fig. 4d). Five hallmark items, including epithelial mesenchymal transition, IL6-JAK-STAT3 signaling, TNFα signaling via NFκB, inflammatory response, and IL2-STAT5 signaling, showed significantly differential enrichment in *SGSM1* low expression phenotype; none in *SGSM1* high expression phenotype (Fig. 4e). These results indicated the potential role of *SGSM1* in tumor microenvironment and immune responses which were critically important in LGG patients.

### Immune infiltration analyses in LGG

**Fig. 5:**
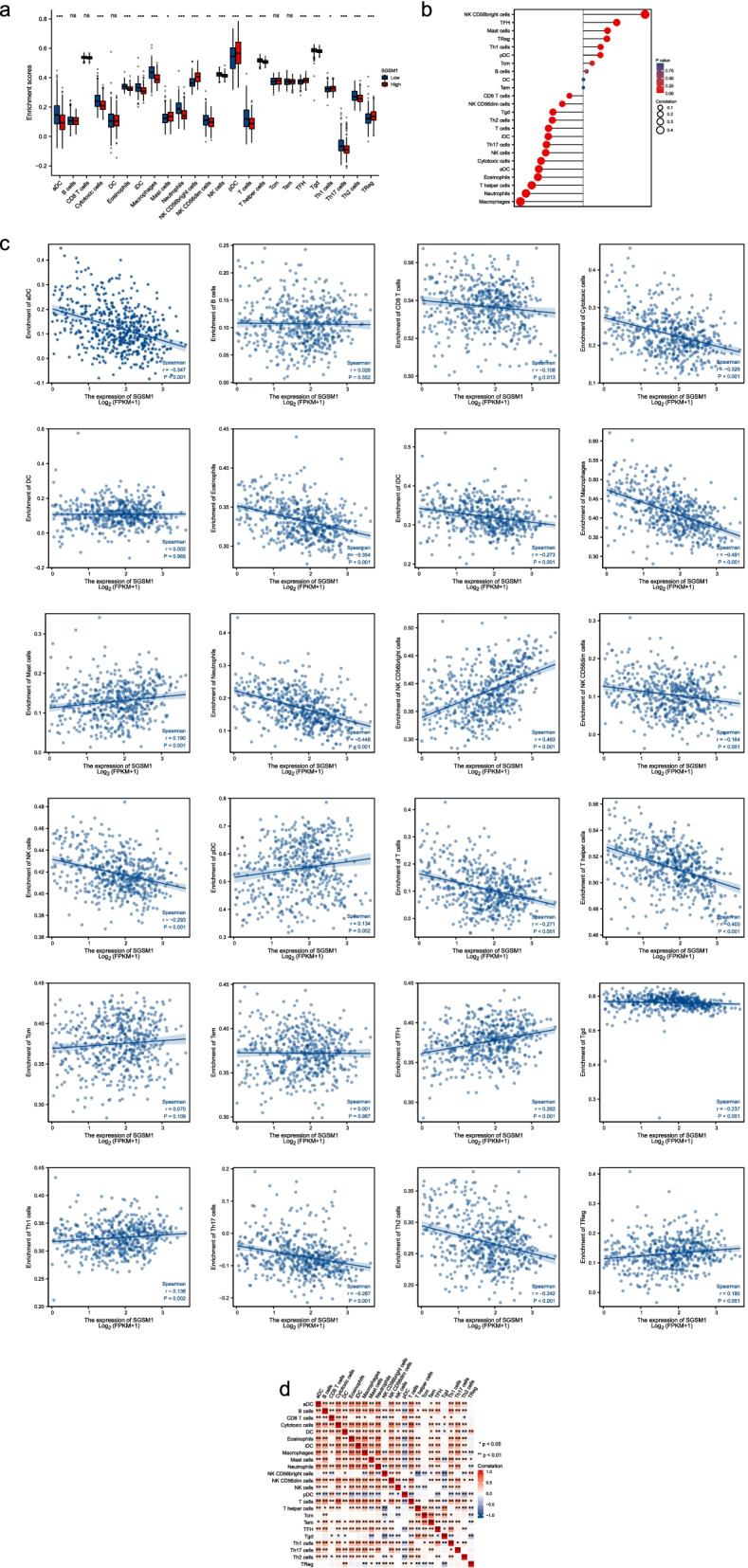
Association between *SGSM1* expression and immune infiltration in LGG. **a** The infiltrating levels of 24 subtypes of immune cells in high and low *SGSM1* expression groups. **b** Correlation between *SGSM1* expression and 24 immune cells. **c** Correlation between *SGSM1* expression and immune infiltration levels. **d** Heatmap of 24 immune infiltration cells in LGGs

Tumor immune infiltration played an important role in the prediction of OS rates. The proportions of 24 subtypes of immune cells in different *SGSM1* expression groups have shown that mast cells (*P* = 0.011), NK CD56bright cells (*P* < 0.001), TFH (T follicular helper, *P* < 0.001), Th1 cells (*P* = 0.042), TReg (*P* < 0.001), and pDCs (plasmacytoid dendritic cells, *P* = 0.001) were significantly increased in high *SGSM1* group, while aDCs (activated DCs, *P* < 0.001), cytotoxic cells (*P* < 0.001), eosinophils (*P* < 0.001), iDCs (immature DCs, *P* < 0.001), macrophages (*P* < 0.001), neutrophils (*P* < 0.001), NK CD56dim cells (*P* = 0.001), NK cells (*P* < 0.001), T cells (*P* < 0.001), Tgd (T gamma delta, *P* < 0.001), T helper cells (*P* < 0.001), Th17 cells (*P* < 0.001), and Th2 cells (*P* < 0.001) were significantly decreased (Fig. 5a).

Moreover, the results have shown positive correlations between *SGSM1* expression and infiltrating levels of mast cells (*r* = 0.190, *P* < 0.001), NK CD56bright cells (*r* = 0.483, *P* < 0.001), pDC (*r* = 0.134, *P* = 0.002), TFH (*r* = 0.262, *P* < 0.001), and Th1 cells (*r* = 0.136, *P* = 0.002). The negative correlations were found between the *SGSM1* expression and infiltrating levels of aDCs (*r* =  − 0.347, *P* < 0.001), CD8 T cells (*r* =  − 0.108, *P* = 0.013), cytotoxic cells (*r* =  − 0.329, *P* < 0.001), eosinophils (*r* =  − 0.354, *P* < 0.001), iDCs (*r* =  − 0.273, *P* < 0.001), macrophages (*r* =  − 0.491, *P* < 0.001), neutrophils (*r* =  − 0.448, *P* < 0.001), NK CD56dim cells (*r* =  − 0.164, *P* < 0.001), NK cells (*r* =  − 0.293, *P* < 0.001), T cells (*r* =  − 0.271, *P* < 0.001), T helper cells (*r* =  − 0.403, *P* < 0.001), Tgd (T gamma delta, *r* =  − 0.237, *P* < 0.001), Th2 (*r* =  − 0.242, *P* < 0.001), Th17 (*r* =  − 0.287, *P* < 0.001), and Treg (*r* = 0.185, *P* < 0.001) (Fig. 5b, 5c). We assessed the possible correlations between the 24 types of immune cells. The heat map has shown that the ratios of different tumor-infiltrating immune cells subtypes were weakly to moderately correlated (Fig. 5d).

**Fig. 6:**
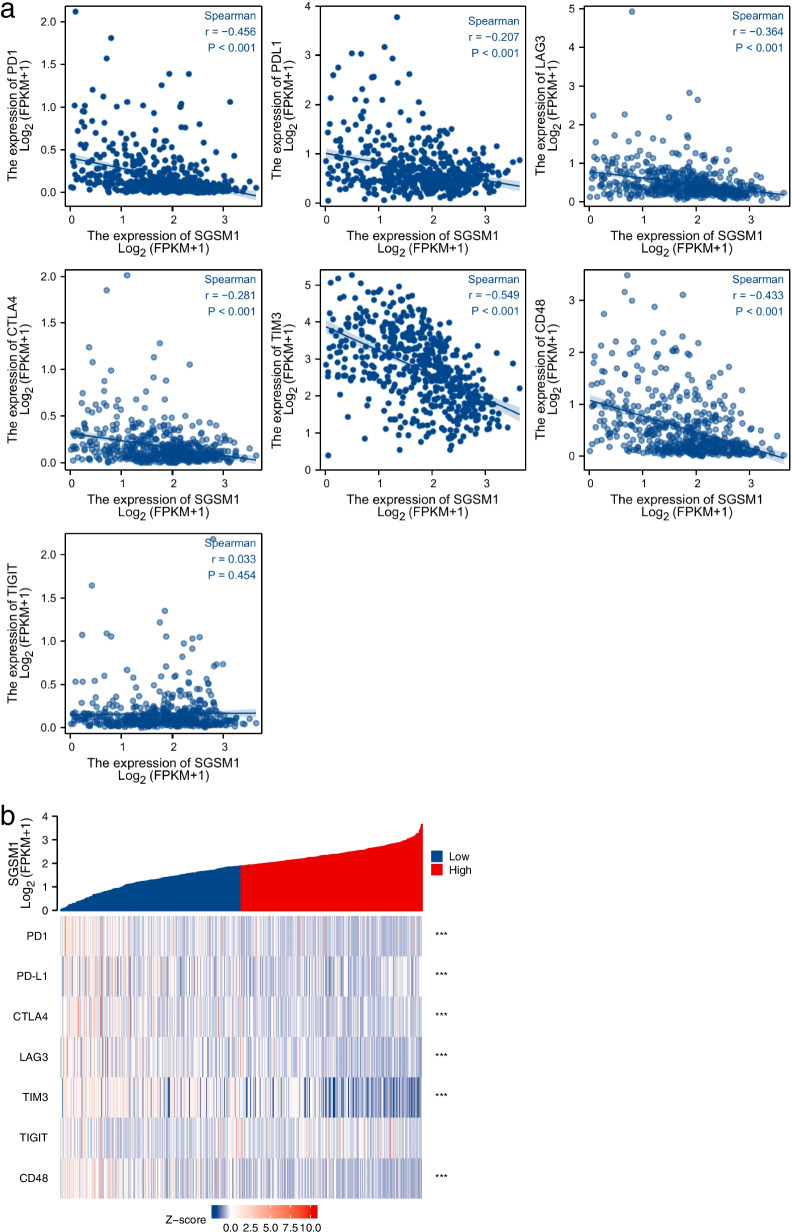
Association between *SGSM1* expression and immune checkpoints. **a** Correlation between *SGSM1* expression and 7 immune checkpoints. **b** Heat map of the immune checkpoints

Furthermore, the association between *SGSM1* expression and immune checkpoints, including PD1, PD-L1, CTLA4, LAG-3, TIM3, TIGIT, and CD48 were analyzed (Fig. 6a). The expression level of PD1, PD-L1, CTLA4, LAG-3, TIM3, and CD48 was negatively correlated with *SGSM1* expression (*P* < 0.001 for all). And the expression level of PD1, PD-L1, CTLA4, LAG-3, TIM3, and CD48 was higher in low *SGSM1* expression group than that in high *SGSM1* expression group (*P* < 0.001 for all, Fig. 6b).

### Association between *SGSM1* expression and clinical features

The main clinical features between low and high *SGSM1* expression groups in LGGs were analyzed (Table 1). In high-expression group, the ratio of WHO grade II (*P* < 0.001), IDH mutation (*P* < 0.001), and 1p/19q codeletion (*P* < 0.001) cases was significantly higher than low-expression group.

**Table 1:** Association between *SGSM1* expression and clinicopathologic features in LGGs

Characteristic	Low *SGSM1* expression	High *SGSM1* expression	*P* value
Age, n (%)			0.338
≤ 40	126 (47.7%)	138 (52.3%)	
> 40	138 (52.3%)	126 (47.7%)	
Gender, n (%)			0.861
Female	118 (44.7%)	121 (45.8%)	
Male	146 (55.3%)	143 (54.2%)	
WHO grade, n (%)			< 0.001*
G2	86 (36.9%)	138 (59.0%)	
G3	147 (63.1%)	96 (41.0%)	
IDH status, n (%)			< 0.001*
WT	80 (30.4%)	17 (6.5%)	
Mut	183 (69.6%)	245 (93.5%)	
1p/19q codeletion, n (%)			< 0.001*
Codel	33 (12.5%)	138 (52.3%)	
Non-codel	231 (87.5%)	126 (47.7%)	

*WT* Wild type, *Mut* Mutant, *Codel* Codeletion, *Non-codel* Non-codeletion

^*^P < 0.05, significant difference

**Fig. 7:**
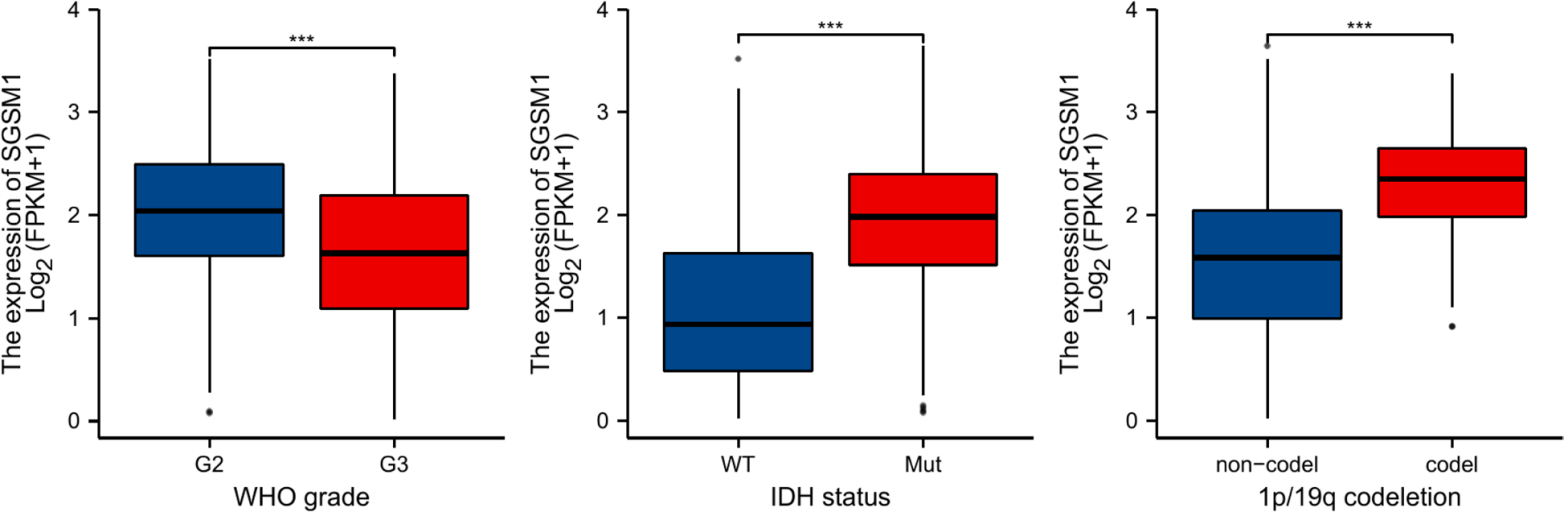
Association between *SGSM1* expression and clinical features

Moreover, we evaluated the *SGSM1* expression level with different clinical characteristics (Fig. 7). The results showed that *SGSM1* was significantly down-regulated in WHO grade III group (*P* < 0.001), IDH wild-type group (*P* < 0.001), and 1p/19q non-codeletion group (*P* < 0.001).

### Relationship between *SGSM1* expression and prognosis

**Fig. 8:**
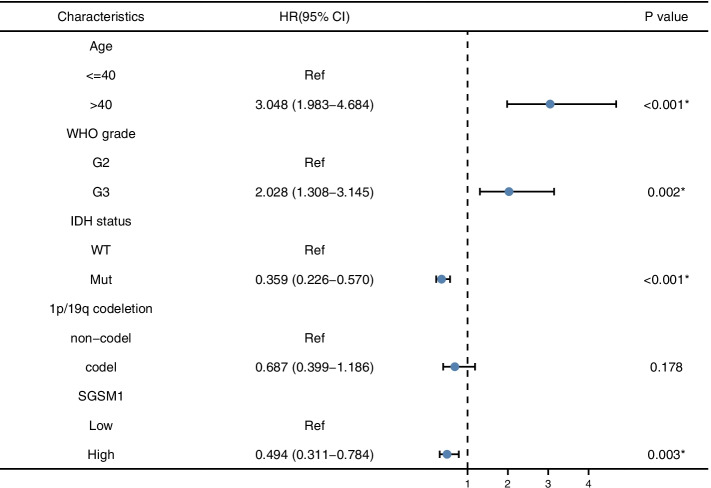
Multivariate Cox analysis of *SGSM1* and other clinicopathological variables

**Fig. 9:**
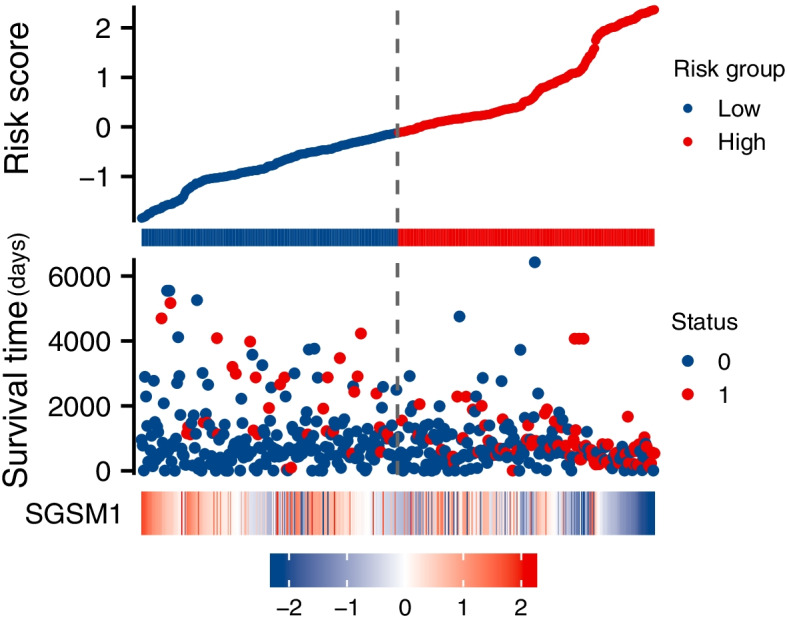
*SGSM1* expression, risk score and survival time distribution

We analyzed the potential predictors by Cox regression analyses, including age, gender, WHO grade, IDH1 status, 1p/19q status, and *SGSM1* expression level. The univariate analysis showed that age, WHO grade, IDH1 status, 1p/19q status, and *SGSM1* expression level were significantly associated with the OS (*P* < 0.001 for all, Table 2). These risk factors were further included in multivariate Cox regression (Fig. 8). The results suggested that *SGSM1* was an independent prognostic factor (HR = 0.494, 95%CI = 0.311–0.784, *P* = 0.003). Then we analyzed the correlation between risk score, survival time, and *SGSM1* expression profiles (Fig. 9).

**Table 2:** Univariate Cox regression analysis of OS in LGGs

Characteristics	Univariate Analysis	
	HR (95% CI)	*P* value
Age		
≤ 40	Reference	< 0.001*
> 40	2.889 (2.009–4.155)	
Gender		
Female	Reference	0.499
Male	1.124 (0.800–1.580)	
WHO grade		
G2	Reference	< 0.001*
G3	3.059 (2.046–4.573)	
IDH status		
WT	Reference	< 0.001*
Mut	0.186 (0.130–0.265)	
1p/19q codeletion		
non-codel	Reference	< 0.001*
codel	0.401 (0.256–0.629)	
SGSM1		
Low	Reference	< 0.001*
High	0.286 (0.193–0.425)	

*WT* Wild type, *Mut* Mutant, *Codel* Codeletion, *Non-codel* Non-codeletion, *HR* Hazard ratio, *CI* Confidence interval

^*^P < 0.05, significant difference

**Fig. 10:**
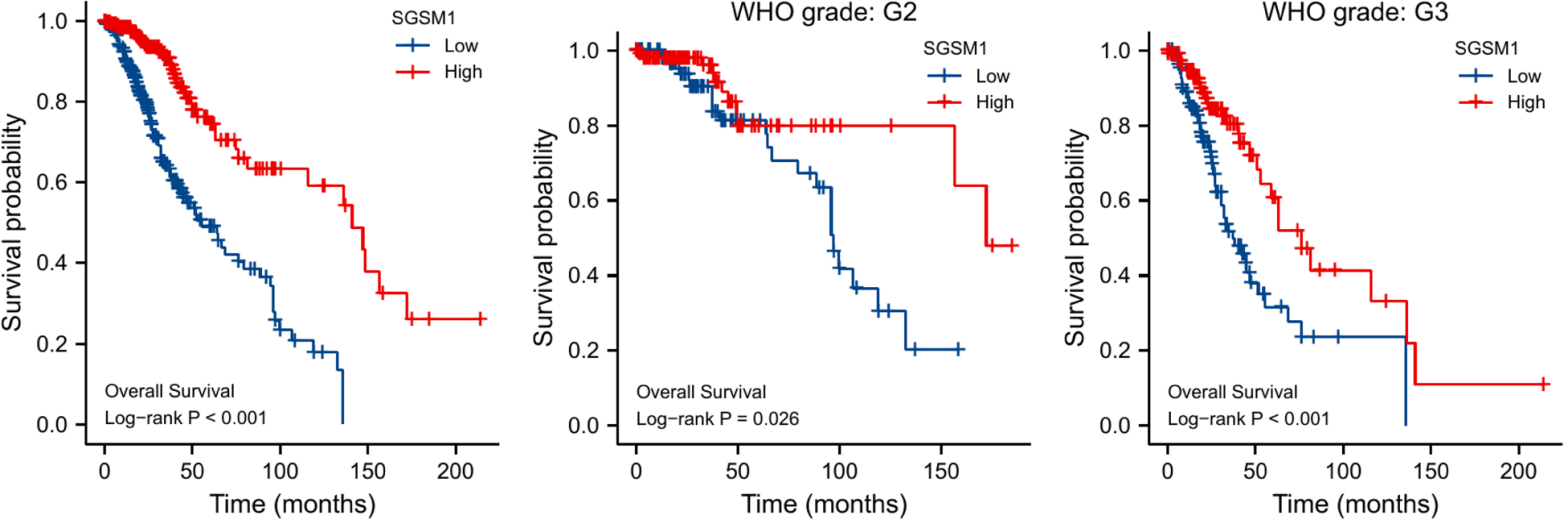
Kaplan–Meier survival analyses of LGG and its subtypes with different *SGSM1* expression levels

Kaplan–Meier analyses showed the relationship between *SGSM1* expression and OS of LGG patients (Fig. 10). Patients with high *SGSM1* expression had a significantly better prognosis than those with low *SGSM1* expression (*P* < 0.001). We further performed Kaplan–Meier analysis in the subgroups of WHO grade, and the results showed that high *SGSM1* expression was correlated with better prognosis in grade II (*P* = 0.026) and grade III (*P* < 0.001), respectively.

**Fig. 11:**
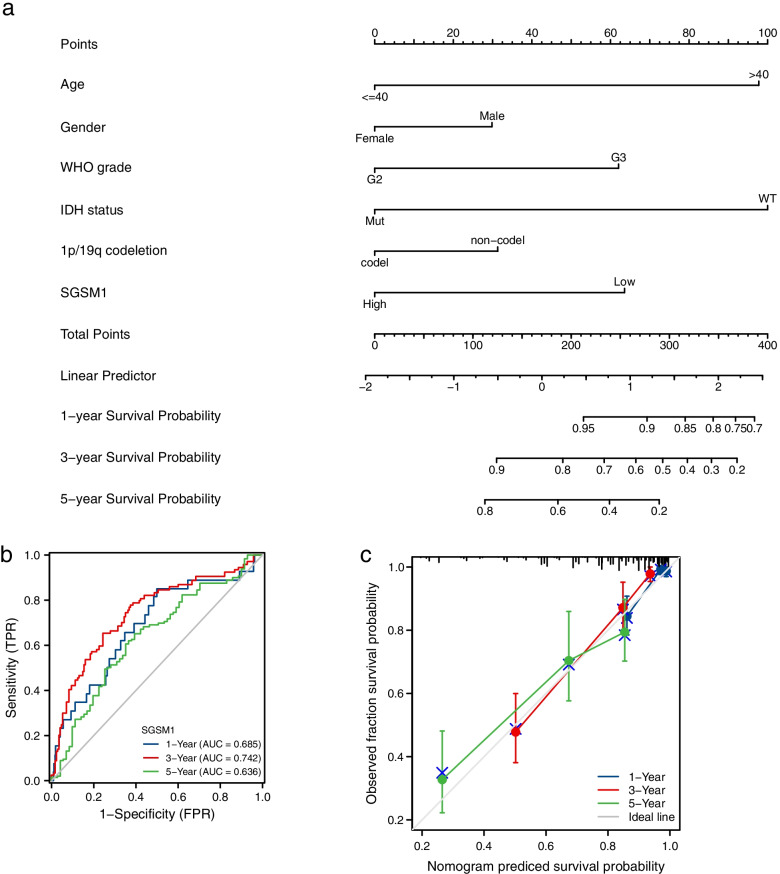
Prognostic prediction model of *SGSM1* in LGGs. **a** Nomogram for 1-year, 3-year and 5-year OS of LGG patients. **b** Time-dependent ROC curves and AUC values for 1-year, 3-year and 5-year OS prediction. **c** Calibration plots for 1-year, 3-year and 5-year OS prediction

The clinical features were integrated into the nomogram model (Fig. 11a), and the C-index was 0.804 (95%CI = 0.779–0.828). We have developed time-dependent ROC curves and calibration plots predicting the probability of 1-year, 3-year, and 5-year OS rates (Fig. 11b). The AUCs in terms of 1-year, 3-year, and 5-year were 0.685, 0.742, and 0.636, respectively. The predicted probability of calibration plots was consistent with the observed results (Fig. 11c).

### Validation of survival analyses

**Fig. 12:**
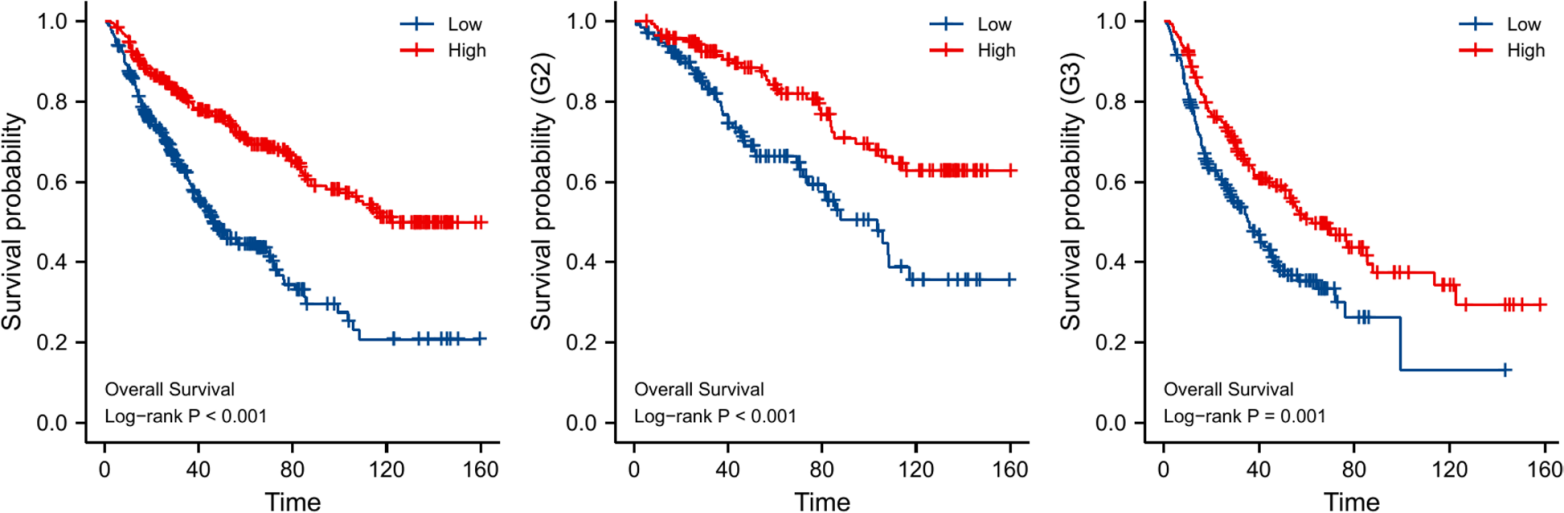
Validation on Kaplan–Meier survival analyses of LGGs, WHO grade II, and III from CGGA database

Using the CGGA database, we validated that *SGSM1* was an independent prognostic factor for LGG prognosis with Cox regression analyses (HR = 0.597, 95%CI = 0.451–0.791, *P* < 0.001, Table 3). We performed the Kaplan–Meier survival analyses in CGGA database (Fig. 12). The results showed that patients with low *SGSM1* expression were correlated with poor outcome in LGG (*P* < 0.001), WHO grade II (*P* < 0.001) and grade III (*P* = 0.001), respectively.

**Table 3:** Validation on Cox regression analyses of OS in LGGs from CGGA database

Characteristics	Univariate analysis		Multivariate analysis	
	HR (95%CI)	*P* value	HR (95%CI)	*P* value
Age				
≤ 40	Reference			
> 40	1.256 (0.978–1.612	0.074		
Gender				
Female	Reference			
Male	0.840 (0.654–1.080)	0.174		
WHO Grade				
G2	Reference		Reference	
G3	2.808 (2.141–3.682)	< 0.001*	2.789 (2.082–3.734)	< 0.001*
IDH status				
WT	Reference		Reference	
Mut	0.428 (0.327–0.561)	< 0.001*	0.706 (0.528–0.944)	0.019*
1p/19q codeletion				
non-codel	Reference		Reference	< 0.001*
codel	0.256 (0.179–0.364)	< 0.001*	0.338 (0.230–0.497)	
SGSM1				
Low	Reference		Reference	< 0.001*
High	0.425 (0.327–0.551)	< 0.001*	0.597 (0.451–0.791)	

*WT* Wild type, *Mut* mutant, *Codel* Codeletion, *Non-codel* Non-codeletion, *HR* Hazard ratio, *CI* Confidence interval

^*^P < 0.05, significant difference

## Discussion

Glioma was the most common type of intracranial malignant tumor [1]. Although LGG was less invasive, the recurrence and malignant progression were almost inevitable even after standard treatment [28]. Thus immunotherapy, gene therapy, and other new therapies have become a promising hope for LGG treatment [29]. It has been necessary to identify prognostic factors to optimize treatment for patients. *SGSM1* was mainly expressed in brain, and it was considered to correlate with small G protein-mediated signal transduction pathway [13]. There were few studies on the potential prognostic role of *SGSM1* in LGGs. Our results have shown *SGSM1* expression was significantly associated with immune infiltration and OS in patients with LGG.

In this study, we first compared *SGSM1* expression in different tumors. The expression of *SGSM1* was significantly down-regulated in most types of cancer, including LGG. Then we analyzed the gene function of *SGSM1* with enrichment analyses. It indicated that *SGSM1* was related to immune response. With the development of tumor microenvironment research, immune cells were considered to play a complex and important role in tumor progression [30–33].

Based on the results of enrichment analyses, we explored the immune infiltration levels by ssGSEA. We found a substantial negative connection of *SGSM1* expression with most immune cells. These immune cells were high infiltrated in low *SGSM1* expression tumors. We considered the excessive immune response and disorganized immune microenvironment contributed to the short survival of these patients [34–36]. Among the immune cells, macrophages (*P* < 0.001) had the highest correlation with *SGSM1* expression, and the infiltration level indicated the prognosis. Increased infiltration of macrophages in low *SGSM1* expression tumors suggested that immune microenvironment was driven from anti-tumor state to immunosuppressive state due to the phenotypic transformation of tumor-associated macrophages, indicated a higher risk of tumor invasion [37]. NK CD56bright cells (*r* = 0.483, *P* < 0.001) were positively correlated with *SGSM1* expression; thus, the infiltration of NK CD56bright cells in tumors was low. NK CD56bright cell had a strong ability to produce cytokines and mainly played an immunomodulatory role [38, 39]. This might lead to the dysregulation of tumor immunosurveillance and anti-tumor effect. Moreover, we revealed the negative correlation between *SGSM1* expression and immune checkpoints, including PD1, PD-L1, CTLA4, LAG-3, TIM3, and CD48. *SGSM1* potentially influenced tumor immunology, and could be a potential therapeutic target for immunotherapy rather than a simple prognostic biomarker. The ratio of WHO grade II, IDH mutation, and 1p/19q co-deletion were significantly higher in the high *SGSM1* expression group. *SGSM1* enhanced in subsets of WHO grade II, IDH mutation, and 1p/19q co-deletion groups. It suggested that *SGSM1* played a potential role in positive prognostic prediction in some way.

Then we analyzed the prognostic role of *SGSM1* in LGG patients. Cox regression analyses showed that *SGSM1* was an independent prognostic factor for LGGs in addition to traditional risk factors, including age, WHO grade, and IDH status. By Kaplan–Meier survival analyses, we found that *SGSM1* expression was correlated to the OS. Low *SGSM1* expression was related to a poor outcome in LGGs, WHO grade II and grade III, respectively. The survival analyses and Cox regression were validated in the CGGA database. The nomogram prognosis model based on *SGSM1* expression level was further established to predict the 1-year, 3-year, and 5-year OS of LGG. The C-index was 0.804 (95%CI = 0.779–0.828). Time-dependent ROC curves and calibration plots illustrated the reliable predictive ability of the nomogram. Our model could provide a new point in outcome prediction and personalized assessment of LGG patients. However, there were still some limitations in this study. Clinical samples should be included for validation. The regulatory mechanism and signaling pathway related to *SGSM1* needed further investigation. The prediction model should be verified in future multicenter studies.

## Conclusion

In summary, *SGSM1* was low expressed in LGGs, and the down-regulation was related to a poor prognosis. Our study has raised a new point of view that *SGSM1* was a promising prognostic factor and a potential therapeutic target for LGGs. Our future study will focus on the mechanism of *SGSM1* in LGGs.

## Data Availability

The public database TCGA and GTEx used in this study were available from UCSC XENA website (https://xenabrowser.net/datapages/). The CGGA data for validation was obtained from CGGA website (http://www.cgga.org.cn).

## References

[1] Brat D, Verhaak R, Aldape K, Yung W, Salama S, Cooper L, Rheinbay E, Miller C, Vitucci M, Morozova O (2015). Comprehensive, integrative genomic analysis of diffuse lower-grade gliomas. N Engl J Med.

[2] Ceccarelli M, Barthel F, Malta T, Sabedot T, Salama S, Murray B, Morozova O, Newton Y, Radenbaugh A, Pagnotta S (2016). Molecular profiling reveals biologically discrete subsets and pathways of progression in diffuse glioma. Cell.

[3] Yang K, Wu Z, Zhang H, Zhang N, Wu W, Wang Z, Dai Z, Zhang X, Zhang L, Peng Y (2022). Glioma targeted therapy: insight into future of molecular approaches. Mol Cancer.

[4] Ostrom QT, Cioffi G, Waite K, Kruchko C, Barnholtz-Sloan JS (2021). CBTRUS statistical report: primary brain and other central nervous system tumors diagnosed in the United States in 2014–2018. Neuro-oncology.

[5] Louis DN, Perry A, Wesseling P, Brat DJ, Cree IA, Figarella-Branger D, Hawkins C, Ng HK, Pfister SM, Reifenberger G (2021). The 2021 WHO classification of tumors of the central nervous system: a summary. Neuro Oncol.

[6] Suzuki H, Aoki K, Chiba K, Sato Y, Shiozawa Y, Shiraishi Y, Shimamura T, Niida A, Motomura K, Ohka F (2015). Mutational landscape and clonal architecture in grade II and III gliomas. Nat Genet.

[7] Jiang T, Mao Y, Ma W, Mao Q, You Y, Yang X, Jiang C, Kang C, Li X, Chen L (2016). CGCG clinical practice guidelines for the management of adult diffuse gliomas. Cancer Lett.

[8] Chen J, Wang Z, Wang W, Ren S, Xue J, Zhong L, Jiang T, Wei H, Zhang C (2020). SYT16 is a prognostic biomarker and correlated with immune infiltrates in glioma: a study based on TCGA data. Int immunopharmacol.

[9] Guo Y, Li Y, Li J, Tao W, Dong W (2021). DNA methylation-driven genes for developing survival nomogram for low-grade glioma. Front Oncol.

[10] Aoki K, Nakamura H, Suzuki H, Matsuo K, Kataoka K, Shimamura T, Motomura K, Ohka F, Shiina S, Yamamoto T (2018). Prognostic relevance of genetic alterations in diffuse lower-grade gliomas. Neuro Oncol.

[11] Shankar GM, Kirtane AR, Miller JJ, Mazdiyasni H, Rogner J, Tai T, Williams EA, Higuchi F, Juratli TA, Tateishi K (2018). Genotype-targeted local therapy of glioma. Proc Natl Acad Sci USA.

[12] Wang Z, Cheng W, Zhao Z, Wang Z, Zhang C, Li G, Wu A, Jiang T. Comparative profiling of immune genes improves the prognoses of lower grade gliomas. Cancer Biol Med*.* 2021. 10.20892/j.issn.2095-3941.2021.0173. 10.20892/j.issn.2095-3941.2021.0173 PMC9088193 34623790

[13] Yang H, Sasaki T, Minoshima S, Shimizu N (2007). Identification of three novel proteins (SGSM1, 2, 3) which modulate small G protein (RAP and RAB)-mediated signaling pathway. Genomics.

[14] Zhang J, Li YQ, Guo R, Wang YQ, Zhang PP, Tang XR, Wen X, Hong XH, Lei Y, He QM (2019). Hypermethylation of SHISA3 promotes nasopharyngeal carcinoma metastasis by reducing SGSM1 stability. Can Res.

[15] Piotrowski A, Koczkowska M, Poplawski AB, Bartoszewski R, Króliczewski J, Mieczkowska A, Gomes A, Crowley MR, Crossman DK, Chen Y (2022). Targeted massively parallel sequencing of candidate regions on chromosome 22q predisposing to multiple schwannomas: an analysis of 51 individuals in a single-center experience. Hum Mutat.

[16] Vivian J, Rao AA, Nothaft FA, Ketchum C, Armstrong J, Novak A, Pfeil J, Narkizian J, Deran AD, Musselman-Brown A (2017). Toil enables reproducible, open source, big biomedical data analyses. Nat Biotechnol.

[17] Goldman MJ, Craft B, Hastie M, Repečka K, McDade F, Kamath A, Banerjee A, Luo Y, Rogers D, Brooks AN (2020). Visualizing and interpreting cancer genomics data via the Xena platform. Nat Biotechnol.

[18] Love MI, Huber W, Anders S (2014). Moderated estimation of fold change and dispersion for RNA-seq data with DESeq2. Genome Biol.

[19] Yu G, Wang LG, Han Y, He QY (2012). clusterProfiler: an R package for comparing biological themes among gene clusters. OMICS.

[20] Kanehisa M, Goto S (2000). KEGG: kyoto encyclopedia of genes and genomes. Nucleic Acids Res.

[21] Subramanian A, Tamayo P, Mootha VK, Mukherjee S, Ebert BL, Gillette MA, Paulovich A, Pomeroy SL, Golub TR, Lander ES (2005). Gene set enrichment analysis: a knowledge-based approach for interpreting genome-wide expression profiles. Proc Natl Acad Sci USA.

[22] Hänzelmann S, Castelo R, Guinney J (2013). GSVA: gene set variation analysis for microarray and RNA-seq data. BMC Bioinformatics.

[23] Bindea G, Mlecnik B, Tosolini M, Kirilovsky A, Waldner M, Obenauf AC, Angell H, Fredriksen T, Lafontaine L, Berger A (2013). Spatiotemporal dynamics of intratumoral immune cells reveal the immune landscape in human cancer. Immunity.

[24] Xu S, Wang Z, Ye J, Mei S, Zhang J (2021). Identification of iron metabolism-related genes as prognostic indicators for lower-grade glioma. Front Oncol.

[25] Liu J, Lichtenberg T, Hoadley KA, Poisson LM, Lazar AJ, Cherniack AD, Kovatich AJ, Benz CC, Levine DA, Lee AV (2018). An integrated TCGA pan-cancer clinical data resource to drive high-quality survival outcome analytics. Cell.

[26] Li K, Chen L, Zhang H, Wang L, Sha K, Du X, Li D, Zheng Z, Pei R, Lu Y (2021). High expression of COMMD7 is an adverse prognostic factor in acute myeloid leukemia. Aging.

[27] Zhao Z, Zhang KN, Wang Q, Li G, Zeng F, Zhang Y, Wu F, Chai R, Wang Z, Zhang C (2021). Chinese Glioma Genome Atlas (CGGA): a comprehensive resource with functional genomic data from chinese glioma patients. Genomics Proteomics Bioinformatics.

[28] Olson JD, Riedel E, DeAngelis LM (2000). Long-term outcome of low-grade oligodendroglioma and mixed glioma. Neurology.

[29] Schiff D, Van den Bent M, Vogelbaum MA, Wick W, Miller CR, Taphoorn M, Pope W, Brown PD, Platten M, Jalali R (2019). Recent developments and future directions in adult lower-grade gliomas: Society for Neuro-Oncology (SNO) and European Association of Neuro-Oncology (EANO) consensus. Neuro Oncol.

[30] Klemm F, Maas RR, Bowman RL, Kornete M, Soukup K, Nassiri S, Brouland JP, Iacobuzio-Donahue CA, Brennan C, Tabar V (2020). Interrogation of the microenvironmental landscape in brain tumors reveals disease-specific alterations of immune cells. Cell.

[31] Guo X, Pan Y, Gutmann DH (2019). Genetic and genomic alterations differentially dictate low-grade glioma growth through cancer stem cell-specific chemokine recruitment of T cells and microglia. Neuro Oncol.

[32] Wu F, Li GZ, Liu HJ, Zhao Z, Chai RC, Liu YQ, Jiang HY, Zhai Y, Feng YM, Li RP (2020). Molecular subtyping reveals immune alterations in IDH wild-type lower-grade diffuse glioma. J Pathol.

[33] Deng X, Lin D, Zhang X, Shen X, Yang Z, Yang L, Lu X, Yu L, Zhang N, Lin J (2020). Profiles of immune-related genes and immune cell infiltration in the tumor microenvironment of diffuse lower-grade gliomas. J Cell Physiol.

[34] Zhang C, Cheng W, Ren X, Wang Z, Liu X, Li G, Han S, Jiang T, Wu A (2017). Tumor purity as an underlying key factor in glioma. Clin Cancer Res.

[35] Chen Q, Han B, Meng X, Duan C, Yang C, Wu Z, Magafurov D, Zhao S, Safin S, Jiang C (2019). Immunogenomic analysis reveals LGALS1 contributes to the immune heterogeneity and immunosuppression in glioma. Int J Cancer.

[36] Cai J, Chen Q, Cui Y, Dong J, Chen M, Wu P, Jiang C (2018). Immune heterogeneity and clinicopathologic characterization of IGFBP2 in 2447 glioma samples. Oncoimmunology.

[37] Aras S, Zaidi MR (2017). TAMeless traitors: macrophages in cancer progression and metastasis. Br J Cancer.

[38] Montaldo E, Vacca P, Moretta L, Mingari MC (2014). Development of human natural killer cells and other innate lymphoid cells. Semin Immunol.

[39] Michel T, Poli A, Cuapio A, Briquemont B, Iserentant G, Ollert M, Zimmer J (2016). Human CD56bright NK Cells: an update. J Immunol.

